# Scientific Items

**Published:** 1879-03-20

**Authors:** 


### SCIENTIFIC ITEMS.

**A Stoppage of the Falls of Niagara.**—The following remarkable account of the stoppage of the Falls of Niagara appeared in the Niagara Mail at the time of the occurrence :

"That mysterious personage, the oldest inhabitant, has no recollection of so singular an occurrence as took place at the Falls on the 30th of March, 1847. The six hundred and twenty thousand tons of water each minute nearly ceased to flow, and dwindled away into the appearance of a mere mill-dam. The rapids above the Falls disappeared, leaving scarcely enough on the American side to turn a grindstone. Ladies and gentlemen rode in carriages one-third of the way across the river towards Canada shore over solid rock as smooth as a kitchen floor. The *Iris* says : 'Table Rock, with some two hundred yards more, was left dry ; islands and places where the foot of man never dared to tread have been visited, flags placed upon them, and mementoes brought away. This unexpected event is attempted to be accounted for by an accumulation of ice at the lower extremity of Fort Erie, which formed a sort of dam between Fort Erie and Buffalo.' "—*Ex.*

**Where the Sun Jumps a Day.**—Chatham Island, lying off the coast of New Zealand, in the South Pacific Ocean, is peculiarly situated, as it is one of the habitable points of the globe where the day of the week changes. It is just in the line of demarcation between dates. There high twelve on Sunday, or noon, ceases, and instantly Monday meridian begins. Sunday comes into a man's house on the east side, and becomes Monday by the time it passes out the western door. A man sits down to his noonday dinner on Sunday, and it is Monday noon before he finishes it. There Saturday is Sunday, and Sunday is Monday, and Monday becomes suddenly transferred into Tuesday. It is a good place for people who have lost much time, for by taking an early start they can always get a day ahead on Chatham Island. It took philosophers and geographers a long time to settle the puzzle of where Sunday noon ceased and where Monday noon began, with a man traveling west fifteen degrees an hour, or with the sun. It is to be hoped that the next English arctic expedition will settle the other mooted question, "Where will one stop who travels northwest continually ?"—*Exchange.*

**The Constitution of Matter in the Gaseous State.**—The development of the kinetic theory of gases covers an enormous advance toward the solution of the great problem of the constitution of matter, a subject, withal, of extreme importance just at this time, in view of Mr. Lockyer's reported resolution of the problem. Whether all matter is proved to be fundamentally one, as Mr. Lockyer asserts, or of several irreducible elements, as chemists have believed hitherto, the discoveries made during recent years with regard to the constitution of gases will mark an era not only in the history of chemistry, but in the wider history of human effort to penetrate the secrets of nature.—*Scientific American.*

**Sanitary Uses of Gunpowder.**—A correspondent writes us from the Sandwich Islands saying that during a long life spent in tropical fever districts, he has been able to escape infection and miasma by the use of gunpowder, supplemented by a few simple precautions against sudden changes of temperature, sunstroke, bad water,. and the like. He uses no water that has not been boiled and afterwards kept from air contact; but his main reliance is upon the practice of burning a thimbleful of gunpowder in his bedroom and very small quantities in his trunk, wardrobe, etc., so as to keep his clothes in an atmosphere feebly charged with gunpowder gas. In Madagascar, Reunion, Mauritius, the East Coast of Tropic Africa, and other fever-smitten lands, he has found such simple means a sure preventive of epidemic and endemic diseases, and has thereby been often brought to the philosophic reflection that gunpowder is destined to invert the aim intended by its fabrication.—*Scientific American.*

**The Struggle for Existence.**—A recent writer on horticulture describes the struggle for life among the plants. He says each plant endeavors, almost unconsciously, to destroy his neighbor, to occupy his ground, to feed upon his nutriment, to devour his substance. There are armies and invasions of grasses, barbarian inroads and extirpations. Every inch of ground is contested by the weeds; the forest is a struggle for precedence; the wars of the roses are a perennial feud. The severest landscape, the stillest woodland, are the mortal arena of vegetable and animal conflict. It is a curious fact that the English plants sent to Australia always kill out the native plants of the same character.—*Exchange.*

**Influence of Sun Spots.**—The theory that the oscillations of the magnetic needle vary according to the increase and decrease of the sun spots has been recently called in question both in England and France. There is a difficulty in the fact that a complete cycle of magnetic change has an average duration of 10.45 years, while the sun spot period appears to be 11.11. Yet it has been claimed and believed by eminent scientists that the sun spots influence the storms and consequently the number of wrecks of each year. The rain fall and amount of grain raised, and even commercial crises have been traced to their influence.

**Wonders of the Atmosphere—Effects of Frost.**—During one of Franklin's voyages the winter was so severe, near the Coppermine river, that the fish froze as they were taken out of the nets. In a short time they became as solid as ice and were easily split open by a blow from a hatchet. If in the completely frozen state they were thawed before the fire, they revived. This is a very remarkable instance of how completely animation is sometimes found suspended in the case of cold-blooded animals.—*Ex.*

**The Human Population of the Globe** is now about 1,439,145,300. Europe contains 312,398,480; Asia, 831,000,000; Africa, 205,210,500; Australia and Polynesia, 4,413,000; America, 86,116,000. This gives an average of 500 inhabitants per square mile of the surface of the globe.

